# From microRNA to protein, linking the neurotrophic hypothesis of depression to the Wistar Kyoto rat

**DOI:** 10.1016/j.nsa.2023.101131

**Published:** 2023-08-19

**Authors:** Erik Kaadt, Natasha Krickau Hedemann, Christian Kroun Damgaard, Heidi Kaastrup Müller, Betina Elfving

**Affiliations:** a Translational Neuropsychiatry Unit, Department of Clinical Medicine, Aarhus University, Palle Juul-Jensens Boulevard 11, A601/A701, 8200, Aarhus N, Denmark; b Department of Molecular Biology and Genetics, Aarhus University, Universitetsbyen 81, Building 1874, 8000, Aarhus C, Denmark

**Keywords:** Bdnf, TrkB, Depression, miRNA, Wistar Kyoto

## Abstract

Animal models of depression offer an alternative research-platform to clinical studies where limitations such as incomplete medical histories, patient heterogeneity, and difficulty tracking comorbidities leading to depression can be more easily overcome. In that context the Wistar Kyoto (WKY) rat strain has for many years been used as an endogenous model of depression. Here we want to further explore the depressive-like phenotype of the WKY rats to extend its validity as a preclinical model of depression.

For this purpose, 49 microRNAs (miRNAs), which have been linked to the neurotrophic hypothesis of depression were selected and their levels were examined in hippocampus, prefrontal cortex, and blood from the WKY rats. Subsequently, miRNA/target mRNA/protein relationships were explored.

Seventeen miRNAs across brain and blood exhibited significant regulation (>30%). Fifteen of 17 miRNAs were upregulated in the WKY rats, when compared to the Wistar Hannover Galas rats. This was coupled with general downregulation of corresponding mRNA targets in both brain regions. We observed decreased *Trkb* mRNA levels in both brain regions, which was coupled with increased miR-103-1 and miR-212 levels (targeting *Trkb*).

While the relationship between mRNA and protein was not entirely linear, we did observe downregulation of TrkB protein in the prefrontal cortex and downregulation of fully mature glycosylated TrkB in both brain regions. This may indicate disruption of the BDNF-TrkB pathway in WKY rats.

In conclusion, this study identifies several molecular alterations in brain and blood from the WKY rats and highlights the WKY rat strain as a viable model of depression.

## Highlights

• 49 microRNAs (miRNAs) were examined in brain and blood from the WKY rats.
• 17 miRNAs across brain and blood exhibited significant regulation.
• Downregulation of corresponding mRNA levels were observed in the brain.
• Disruption of the BDNF-TrkB pathway in the WKY rats is suggested.

## Abbreviations

BDNF: brain-derived neurotrophic factor
miRNA: microRNA
WKY: Wistar Kyoto
HIP: hippocampus
PFC: prefrontal cortex
WHO: World Health Organization
ncRNAs: non-protein coding RNAs
AU: adenylate-uridylate
AUBPs: adenylate-uridylate (AU) rich element binding proteins
VEGF: vascular endothelial growth factor
proBDNF: The precursor of BDNF
mBDNF: mature BDNF
MMP: matrix metalloproteinase
TrkB: ropomyosin-related kinase B
CREB: cAMP response element-binding protein
GABA: gamma-aminobutyric acid
HPA: hypothalamic-pituitary-adrenal
NGF: Nerve growth factor
qPCR: quantitative Polymerase Chain Reaction
WHG: Wistar Hannover Galas
ELISA: Enzyme-linked Immnunosorbent Assay
NTC: No Template Control
OBB: Odyssey Blocking Buffer
BSA: bovine serum albumin
CUMS: chronic unpredictable mild stress
EE: Enriched environment
AAV: Adeno-Associated Virus

## 1 Introduction

Depression is a serious mental disorder affecting 280 million people worldwide and is, according to the World Health Organization (WHO), the leading cause of disability and a major contributor to the overall global burden of disease (WHO, 2021). Depression is a multifaceted disease with a complex pathogenesis and despite the prevalence and burden of depression, the understanding of the pathophysiology is rudimentary. Furthermore, a large fraction of depressed patients do not respond adequately to currently available pharmacological treatments (Sinyor et al., 2010; Trivedi et al., 2006).

Clinical studies have contributed significantly to the current understanding of the neurobiology of depression. However, low sample sizes, incomplete medical histories, patient heterogeneity, and difficulty tracking comorbidities leading to depression are common in such studies (Millard et al., 2020; Rajkowska, 2003). To accommodate for these limitations, preclinical models of depression offer an alternative solution to further explore the neurobiology of depression, and to investigate novel treatment strategies (Millard et al., 2020).

The Wistar Kyoto (WKY) rat strain has for many years been used as an endogenous model of treatment resistant depression, as the rats do not respond to fluoxetine, a selective serotonin reuptake inhibitor (Millard et al., 2020). The WKY rat strain is characterized by elevated anxiety- and depressive-like behavior (Kaadt et al., 2021; McAuley et al., 2009), abnormalities in the monoamine system, glutamate and gamma-aminobutyric acid (GABA) malfunction, dysregulation of the hypothalamic-pituitary-adrenal (HPA) axis, and attenuated serum and brain “Brain-derived neurotrophic factor” (BDNF) levels (Aleksandrova et al., 2019; Kyeremanteng et al., 2014).

Here we want to further link WKY rats to the depressive phenotype to extend its validity as a preclinical model of depression.

Several neurobiological abnormalities and genetic factors have consistently been reported to play a role in depression. For decades, the dysregulation of BDNF and more recently, the vascular endothelial growth factor (VEGF) have been linked to the depressive pathophysiology and antidepressant treatment in preclinical and clinical studies (Deyama et al., 2019; Deyama and Duman, 2020; Dwivedi et al., 2003; Elfving et al., 2010; Karege et al., 2005; Levy et al., 2018; Qi et al., 2015; Sheldrick et al., 2017). In addition, substantial evidence has demonstrated that expression of non-coding RNAs such as microRNAs (miRNAs) are altered in brain and blood from depressed patients compared to healthy subjects (Dwivedi, 2014; Lopez et al., 2018; Meerson et al., 2010). As miRNAs regulate the turnover and translation of complimentary mRNA-targets (Bartel, 2009; Krol et al., 2010) it is particularly relevant to further investigate miRNAs targeting protein-coding transcripts, which are fundamental for the depressive pathophysiology (Dwivedi, 2014; Lopez et al., 2018; Meerson et al., 2010).

For this purpose, we selected 49 miRNAs targeting *Bdnf*, the *Bdnf*-receptor *Tropomyosin-related kinase receptor B* (*Trkb*), *Vegf*, and several other molecules involved in the expression and processing of BDNF or the efficiency by which miRNAs elicit their post-transcriptional function (Tables S1 and S2). Aside from miRNA levels we investigated the levels of cognate mRNA- and protein-targets to get a comprehensive overview of the central and peripheral regulation in the WKY rats.

## 2 Experimental procedures

### 2.1 miRNA selection

A combination of literature and online tools (*TargetScan* and *miRTarBase*) was utilized to identify miRNAs, which target the expression of genes related to depression (*Bdnf*, *Vegf*, Nerve growth factor (*Ngf), Metalloproteinase (Mmp)9, Serotonin transporter (Sert), Sortilin, Creb, Trka, Trkb, Trkc, Hur, and Hud*) (Table S1). In addition, NCBI was used to identify miRNAs, previously observed to be dysregulated in depression (keywords; microRNA, miR, BDNF, VEGF, depression, antidepressant, regulation, brain). Initially, 85 miRNAs were selected. LNA-gapmer primers from Exiqon (Denmark) were used for real-time quantitative Polymerase Chain Reaction (qPCR) experiments due to their high sensitivity and specificity. However, the utilization of LNA primers limited the number of miRNAs available for investigation to 49 (Table S2).

### 2.2 Animals

Male Wistar Hannover Galas (WHG) and WKY rats (Taconic Biosciences) (10–12 weeks of age; 280–350g) were cage-housed in pairs (cage 1291H Eurostandard Type III H, 425 x 266 ​× ​185 ​mm, Techniplast, Italy) at 20 ​± ​2 ​°C in a 12 ​h light/dark cycle (lights on at 7.00 a.m.) and with free access to food and water. The WHG rats were used as a control group. Ten rats were included in each group for real-time qPCR and Western blotting studies. All animal procedures were approved by the Danish National Committee for Ethics in Animal Experimentation (2012-15-2934-00254).

### 2.3 Euthanization

The rats were euthanized by decapitation. Whole blood was collected from the neck wound in EDTA tubes and serum was collected in anticoagulant-free tubes with gel. After 1 ​h the serum samples were centrifuged (1550*g*, 10 ​min, 4 ​°C). The brain was removed and hippocampus and prefrontal cortex were dissected and frozen with powdered dry-ice immediately. Brain tissue and blood were stored at -80 ​°C until real-time qPCR analysis, Western blotting, and Enzyme-linked Immnunosorbent Assay (ELISA) studies were conducted.

### 2.4 RNA extraction from brain tissue and blood

Homogenization of left hippocampus and prefrontal cortex was performed with Mixer-Mill (Retsch) (1 ​min, 30Hz). The samples were stored overnight at -80 ​°C and the next day homogenization was repeated (1 ​min, 30Hz). Total RNA was isolated using ABI PRISM^TM^ 6100 Nucleic Acid Prepstation (Applied Biosystems, CA.) according to manufacturer's instructions (RNA Tissue-Filtr-DNA protocol or RNA Blood-DNA/ultra-low gDNA protocol). The RNA concentration and purity (A260/A280 and A260/A230 ratios) were determined by NanoDrop 1000 Spectrophotometer (Thermo Fisher Scientific, Delaware, USA).

### 2.5 miRNA expression levels in brain tissue and whole blood were investigated using real-time qPCR

cDNA synthesis was conducted using the Universal cDNA synthesis kit II (Exiqon, Denmark) and with a final RNA-concentration of 10 ​ng/μL (total RNA). The cDNA samples were diluted 1:30 with nuclease free water (Exiqon, Denmark) before being used as a real-time qPCR template.

Real-time qPCR experiments were carried out in 96-well PCR-plates (SorensonTM Bioscience, Inc.) using the Mx3005P (Stratagene, USA) and SYBR Green (Exiqon, Denmark). Each SYBR Green reaction (10 ​μL total volume) contained 5 ​μL SYBR Green, 1 ​μL primer set (LNA^TM^, Exiqon, Denmark) and 4 ​μL of diluted cDNA. The real-time qPCR set-up included a melting curve and was run according to the recommendation of Instruction manual v6.2 for miRNA (10 ​min 95 ​°C, 40 cycles of 10 sec 95°C, 1 ​min 60 ​°C, 1 ​min 95 ​°C, 30 sec 60°C, 30 sec 95°C). Each plate contained No Template Control (NTC), a standard curve (Standard 1 diluted 1:3, followed by 2-fold dilution Standard 2–5) and samples. NTC, standards, and samples were run in duplicate.

To ensure adequate expression of targets in brain tissue and blood a standard curve was performed for each of the 49 primer sets before running the samples. The amplicons were only included if the melting curve revealed a single product and the amplification efficacy was between 90 and 110%. In brain tissue 31 miRNAs were included, and 22 miRNAs were included in whole blood. Three reference genes were additionally included for all samples based on recommendation from Exiqon (RNU5G, RNU1A1, and U6). However, these genes did not produce a satisfying efficiency (90–110%) in the real-time qPCR experiments and were not included in the normalization procedure. Hence, the 8 most stable expressed miRNAs were identified in hippocampus, prefrontal cortex, and whole blood and a stability comparison was conducted with the NormFinder software (https://moma.dk/software/normfinder). The most stable combinations were miR-125a-5p/miR-185-5p for hippocampus, miR-16-5p/miR-128-3p for prefrontal cortex, and miR-20a-5p/miR-185-5p for whole blood. Values from each individual sample were normalized with the geometric mean of the relevant reference gene combination.

### 2.6 mRNA expression levels in brain tissue were investigated using real-time qPCR

mRNA was reversely transcribed using random hexamers and Superscript IV Reverse Transcriptase (Invitrogen, CA, USA) following manufacturer's instructions. The input RNA concentration in each sample was 22 ​ng/μl for brain tissue. The cDNA samples were diluted 1:10 with DEPC water before being used as a real-time qPCR template.

Real-time qPCR experiments were carried out in 96-well PCR-plates (SorensonTM Bioscience, Inc.) using the Mx3005P (Stratagene, USA) and SYBR Green (Sigma-Aldrich, St. Louis, MO, USA). The expression of 8 different reference genes (*18s rRNA, Actb, CycA, Gapdh, Hmbs, Hprt1, Rpl13A, and Ywhaz)* and 8 target genes (*Bdnf*, *Creb, Hur, Mmp9, Sortilin, Trkb, Trkc, Vegf*) were investigated as previously described (Bonefeld et al., 2008; Du Jardin et al., 2017). The gene specific primer pairs are given in Table S3. Each SYBR Green reaction (10 ​μl total volume) contained 1 ​× ​SYBR Green mastermix (Sigma–Aldrich, St. Louis, MO, USA), 0.5 ​μM primer pairs, and 3 ​μl of diluted cDNA. The thermal conditions for the PCR were 3 ​min at 95 ​°C, followed by 40 cycles of 10 ​s denaturation at 95 ​°C, 30 ​s annealing at 60 ​°C, and 60 ​s extension at 72 ​°C. Each run was completed by dissociation curve analysis to confirm the amplification specificity and absence of primer dimers. We generated a standard curve, performed in duplicate, on each plate. Primers were only included if the melting curve revealed a single product and the amplification efficacy was between 90 and 110%.

For hippocampus and prefrontal cortex stability comparison of the expression of the reference genes was conducted with the NormFinder software (http://moma.dk/software/normfinder). The most stable combination was *Hprt/CycA* for hippocampus and *Hprt/Ywhaz* for the prefrontal cortex, respectively. Values from each individual sample were normalized with the geometric mean of the relevant reference gene combination.

### 2.7 Protein levels in brain tissue were investigated using western blotting

Left hippocampus and prefrontal cortex were homogenized with Mixer-Mill (Retsch) (two times, 1 ​min, 30Hz) in cell lysis buffer (Bio-Rad) and mixed with SDS sample buffer (125 ​mM Tris-HCl (pH 6.8), 20% glycerol, 4% SDS, 0.02% bromophenol blue, 125 ​mM dithiothreitol) to a final concentration of 2 ​μg/μl. Aliquots of lysate (20 ​μg total protein) were separated on 10% criterion TGX gels (Bio-Rad) using Tris-glycine running buffer or on 10% NuPAGE Bis-Tris gels (Invitrogen) using a MES-buffer system. Proteins were transferred to nitrocellulose membranes (Bio-Rad) using the Trans-blot Turbo system (Bio-Rad). Blots were blocked in Odyssey Blocking Buffer (OBB, LI-COR) and probed with primary antibodies diluted in OBB containing 0.1% Tween-20 overnight at 4 ​°C (Primary antibodies: rabbit anti-BDNF (1:2000, Sigma AV41970, AB_1,845,423), rabbit anti-CREB (1:500, Cell Signaling #9197, AB_331277), rabbit anti-HuR (1:500, Abcam ab200342, AB_2784506), rabbit anti-Sortilin (1:500, Alomone Labs ANT-009, AB_2040216), goat anti-TrkB (1:500, R&D Systems AF1494, AB_2155264), rabbit anti-MMP9 (1:400, Sigma AV33090, AB_1854045), mouse anti-VEGF (1:250, R&D Systems MAB564, AB_2212820), mouse anti-β-actin (1:2000, LI-COR 926–42212, AB_2756372) and rabbit anti-β-actin (1:2000, LI-COR 926–42210, AB_1850027). After washing in TBST (50 ​mM Tris-HCl, 150 ​mM NaCl, pH 7.6, 0.1% Tween-20), the membranes were incubated with the appropriate IRDye conjugated secondary antibody for 1 ​h at RT: IRDye 800CW goat anti-rabbit IgG, IRDye 680RD goat anti-rabbit IgG, IRDye 800CW goat anti-mouse IgG, IRDye 680RD goat anti-mouse IgG or IRDye 800CW donkey anti-goat IgG at 1:15,000 dilution (LI-COR). Infrared signals were detected using the Odyssey CLx infrared imaging system, and bands were quantified using Image Studio software (LI-COR). β-actin levels did not differ between groups and was used as loading control to normalize band intensities in all blots.

### 2.8 BDNF protein levels in serum were investigated using ELISA

The concentrations of mBDNF and proBDNF were determined using ELISA kits (R&D systems, Minnesota, USA). The standard curves and the samples were run in duplicates. The samples were diluted 1:20 in PBS+1% bovine serum albumin (BSA) for mBDNF measurements and undiluted for proBDNF measurements. The standard curve for mBDNF was prepared in PBS+1%BSA (ranging from 1133.3 to 17.2 ​pg/ml) and the standard curve for proBDNF was prepared in PBS-Tween20 (ranging from 5000 to 4.9 ​pg/ml). 96-well immunoplates (NUNC, Denmark) were used and the experiments were run according to the distributor's recommendation. The reactions were stopped by adding 1M H_2_SO_4_ and the absorbance's were immediately measured at 450 ​nm and 540 ​nm (EL 800 Universal Microplate reader, Bio-Tek Instruments Inc, USA).

### 2.9 Statistics

All statistical analysis was performed using GraphPad Prism version 6.00 for Windows (GraphPad Software, La Jolla California USA). Results were analyzed for paired significance using Students T-test (two-tailed, assuming equal SD). Prior to analysis all groups were checked for normality by the D'Agostino & Pearson omnibus normality test. A p-value <0.05 was considered to be statistically significantly different. For further evaluation of the data adjusted p-values using the Benjamini-Hochberg (BH) correction for multiple testing were included.

## 3 Results

### 3.1 miRNA expression in hippocampus, prefrontal cortex, and whole blood

**Fig. 1:**
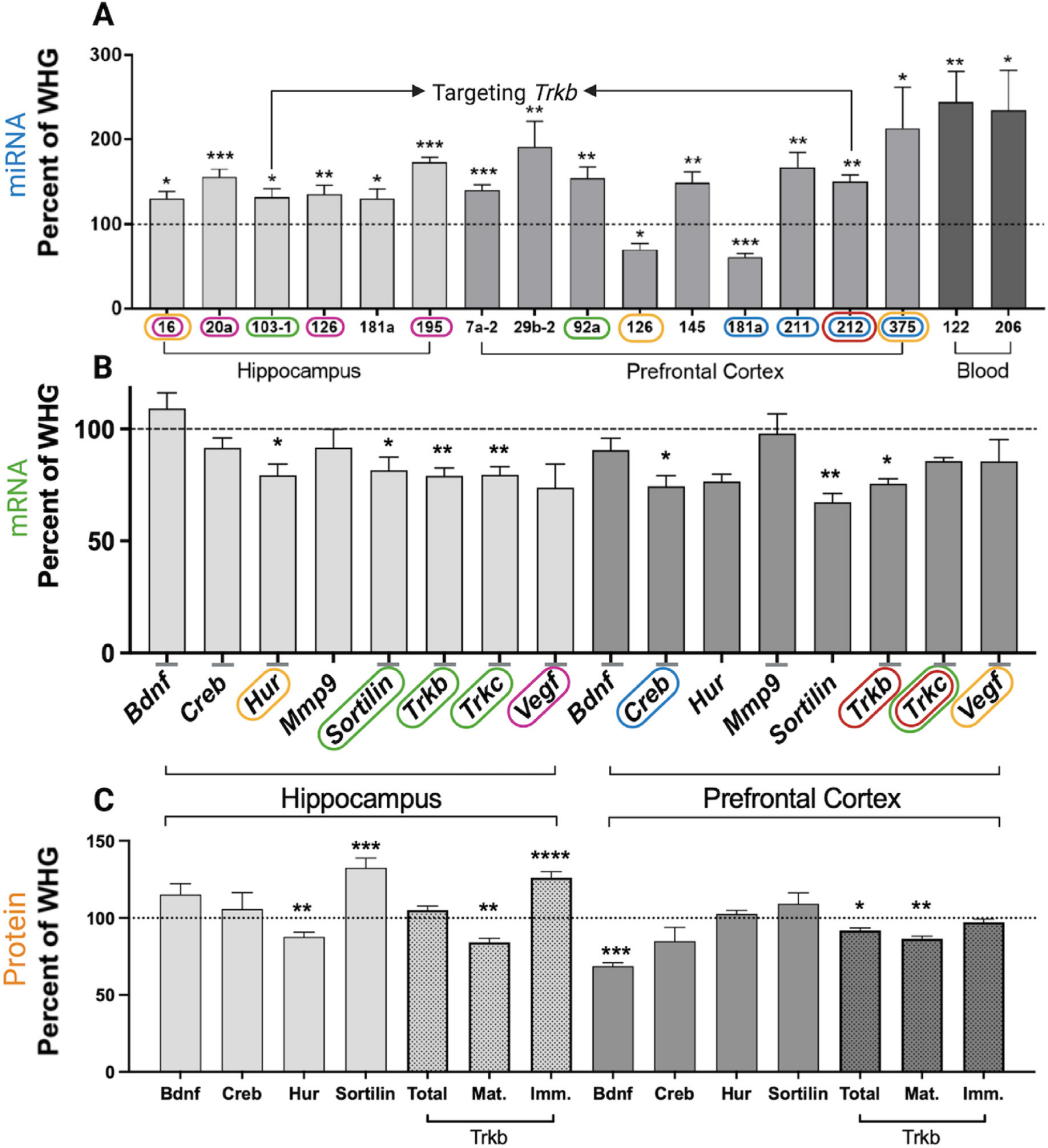
A: Real-time qPCR: Significantly dysregulated (>30% regulation) miRNAs in the hippocampus, prefrontal cortex, and whole blood from the WKY rats compared to the WHG rats (*t*-test, ∗p ​< ​0.05, ∗∗p ​< ​0.01, ∗∗∗p ​< ​0.001). The expression of WHG has been set to 100% (horizontal line) and the values are given in mean ​+ ​SEM (n ​= ​10 in each group). Specific values are included in table S4-6. B: Real-time qPCR: Expression of selected mRNA targets in hippocampus and prefrontal cortex from WKY rats compared to WHG rats (*t*-test, ∗p ​< ​0.05, ∗∗p ​< ​0.01). The expression of WHG has been set to 100% (horizontal line) and the values are given in mean ​+ ​SEM (n ​= ​10 in each group). miRNAs targeting selected mRNAs has been marked with color codes. Specific values are included in table S7. C: Western blotting: Expression of selected proteins in hippocampus and prefrontal cortex from WKY rats compared to WHG rats (*t*-test, ∗p ​< ​0.05, ∗∗p ​< ​0.01, ∗∗∗p ​< ​0.001, ∗∗∗∗p ​< ​0.0001). The expression of WHG has been set to 100% (horizontal line) and the values are given in mean ​+ ​SEM (n ​= ​10 in each group, except WKY hippocampus n ​= ​9). Blots are included in Figure S1 and S2. Specific values are included in table S8. Abbreviations: miRNA – microRNA; WKY – Wistar Kyoto; WHG - Wistar Hannover Galas. (For interpretation of the references to color in this figure legend, the reader is referred to the Web version of this article.)

Thirty-one miRNAs were expressed at a detectable level in hippocampus (Table S4) and prefrontal cortex (Table S5), and 22 miRNAs were expressed in whole blood (Table S6) in the WHG/WKY rats. Significance of miRNAs was determined by a dual criterion consisting of a p-value below 0.05 and a mean regulation of minimum 30%. Six, nine, and two miRNAs fulfilled the dual criteria in the WKY rats compared to the WHG rats in hippocampus, prefrontal cortex, and whole blood, respectively (Fig. 1A and Tables S4–S6). miR-126 and miR-181a were both up-regulated in hippocampus and down-regulated in prefrontal cortex. Fifteen of the 17 dysregulated miRNAs exhibited significant upregulation in the WKY rats compared to the WHG rats (Fig. 1A). After multiple correction 13 out of 17 miRNAs remained significantly dysregulated in the WKY rats compared to the WHG rats. MiR-103-1 and miR-181a in hippocampus, miR-375 in prefrontal cortex, and miR-206 in whole blood were no longer significantly regulated in the WKY rats compared to the WHG rats.

### 3.2 Investigation of mRNA-targets of dysregulated miRNAs in brain tissue

To examine whether the overall general upregulation of miRNAs in the brain resulted in corresponding downregulation of matched mRNA-targets, the expression of eight mRNA targets were investigated (*Bdnf*, *Creb, Hur, Mmp9, Sortilin, Trkb, Trkc, Vegf)*. The results are given in Fig. 1B and Table S7. The six miRNAs upregulated in hippocampus were predicted to target *Bdnf, Creb, Hur, Sortilin, Trkb*, *Trkc*, *and Vegf*, while the 7 miRNAs upregulated in prefrontal cortex target *Bdnf, Creb, Mmp9, Trkb, Trkc, and Vegf (*Fig. 1B – gray horizontal lines - selected individual miRNA/mRNA relationships are shown in color coded circles). In agreement with this prediction we observed significant downregulation of *Hur, Sortilin, Trkb*, *Trkc*, and a tendency for downregulation of *Vegf* (p ​= ​0.06) in the hippocampus, while in prefrontal cortex we observed significant downregulation of *Creb*, *Sortilin, Trkb,* and a tendency of downregulation for *Trkc* (p ​= ​0.07). None of the mRNA-targets were upregulated. After multiple correction four out of seven mRNAs remained significantly dysregulated in the WKY rats compared to the WHG rats. *Sortilin* in hippocampus, and *Creb* and *Trkb* in prefrontal cortex were no longer significantly regulated in the WKY rats compared to the WHG rats.

### 3.3 Investigation of proteins associated to dysregulated mRNAs in brain tissue

To further explore the mRNA/protein relationships, we investigated the protein levels of BDNF, CREB, HuR, MMP9, Sortilin, TrkB, and VEGF (Fig. 1C, Figs. S1–S2, and Table S8). VEGF and MMP9 exhibited low expression/degradation and the results were excluded. In the hippocampus, HuR was down-regulated and Sortilin was up-regulated and in the prefrontal cortex, BDNF was down-regulated in the WKY rats compared to the WHG rats (Fig. 1C). Total TrkB levels were unaltered in hippocampus and downregulated in prefrontal cortex (Fig. 1C). However, the fully mature glycosylated form of TrkB (Mat.) was significantly down-regulated in both brain regions, while the immature glycosylated form of TrkB (Imm.) was up-regulated in the hippocampus in the WKY rats compared to the WHG rats (Fig. 1C). After multiple correction six out of seven proteins remained significantly dysregulated in the WKY rats compared to the WHG rats. Total TrkB in prefrontal cortex was the only protein no longer significantly regulated in the WKY rats compared to the WHG rats.

### 3.4 Investigation of BDNF serum protein levels

In whole blood miR-122 and miR-206 were significantly up-regulated to 244% and 234% in the WKY rats compared to the WHG rats, respectively (Fig. 1A). As both miRNAs target *Bdnf* we decided to investigate proBDNF and mature BDNF (mBDNF) in serum. However, the proBDNF levels were below the detection limit and the mBDNF levels were identical in the WHG and the WKY rats (100.0 ​± ​19.8% vs 102.3 ​± ​19.4%, mean ​± ​SEM, p ​= ​0.9357).

## 4 Discussion

The use of animal models in depression research are crucial to delineate the underlying neurobiological and molecular mechanisms. In this study, we utilized the endogenous WKY rat model of depression to investigate the expression of pre-selected miRNAs and corresponding mRNA- and protein-targets in core brain areas of depression and blood. We generally observed increased levels of the selected miRNAs in both brain areas, which were accompanied by a general tendency for downregulation of their corresponding mRNA targets. miR-16 (targets *Hur*) and miR-103-1 (targets *Sortilin, Trkb,* and *Trkc*) exhibited an inverted relationship with their corresponding mRNA targets in the hippocampus. In the prefrontal cortex, miR-181a, miR-211, miR-212, and miR-375 all target *Creb*, which was significantly down-regulated. miR-212 also targets *Trkb*, which is also down-regulated at the mRNA level.

In accordance with these observations, miR-16 has previously been reported to be increased in the hippocampus from rats subjected to maternal deprivation (MD), an early life stress model of depression (Bai et al., 2012) and knockdown of miR-16 in the hippocampus of mice using antagomiRs, leads to an antidepressive effect (Baudry et al., 2010; Launay et al., 2011).

Contrary to our results Bai et al. reported that mRNA and protein levels of BDNF were decreased in the MD rats compared to control rats (Bai et al., 2012). However, in the MD study the rats were subjected to behavioral tests 24 ​h before euthanization and anesthetized with 10% chloral hydrate before being sacrificed. Hence, it cannot be ruled out that this could affect the Bdnf levels. In our study the rats have not been subjected to any kind of behavioral tests and we only observed a significant downregulation of the BDNF protein levels in the prefrontal cortex (Fig. 1C). This is in accordance with a previous study by Vinod et al. (2012) reporting 27% lower BDNF levels in frontal cortex in WKY rats compared to Wistar rats. In the literature, several BDNF-related strain differences between WKY and Wistar rats have been reported. In female rats, loss of left hippocampal volume (Tizabi et al., 2010), reduced BDNF protein levels in hippocampus (Harlan Laboratories, Indianapolis, IN) (Hauser et al., 2011) and ventral striatum (Charles River Laboratory, USA) (Dong et al., 2020) have been observed. In male rats (Charles River, Quebec, Canada), reduced serum BDNF protein levels have been reported, however this difference was not observed in brain tissue (hippocampus, frontal cortex, and neocortex) (Kyeremanteng et al., 2012). This is contrasted by our results, as we observed reduced BDNF protein levels in the prefrontal cortex and no changes in serum BDNF protein levels. The WKY and WHG rats included in our study are from Taconic Biosciences (Denmark), and it could be speculated that WKY rats from different vendors exhibit different deficiencies in the BDNF signaling pathway while maintaining the same phenotype. This is supported by studies displaying different genetic architecture and behavioral outputs from WKY rats between vendors (Paré and Kluczynski, 1997; Zhang-James et al., 2013).

Therefore, to further explore the BDNF signaling, we investigated the mRNA expression and protein levels of the BDNF receptor TrkB (Fig. 1B and C). The *Trkb* mRNA level was down-regulated in both brain regions, which indicates diminished BDNF signaling despite unaltered BDNF levels in the hippocampus. At the protein level, total TrkB was downregulated in prefrontal cortex, but unaltered in the hippocampus. However, significantly lower amounts of mature glycosylated TrkB were observed in both brain regions. This indicate that mechanisms other than regulation of the transcript levels imposed by miRNAs are involved in the coordinated reduction of BDNF signaling. Factors involved in TrkB maturation could potentially be affected by a subset of the dysregulated miRNAs.

While the BDNF-TrkB pathway is generally reported to promote neuronal survival, growth, and differentiation, it has been reported that pro-BDNF induce neuronal apoptosis when activating a receptor complex with p75NTR and Sortilin (Teng et al., 2005). To further explore BDNF signaling pathways in the WKY rats, the expression level of both Sortilin mRNA and protein (Fig. 1A and B) were investigated. While *Sortilin* mRNA was down-regulated in both brain regions, the protein levels were robustly up-regulated in the hippocampus and unaltered in the prefrontal cortex. We were not able to measure pro-BDNF reliably in the brain by Western blotting, and in serum the level of pro-BDNF was below detection limit of the ELISA kit.

It can be speculated that neuronal survival and growth is compromised through the diminished BDNF signaling due to TrkB downregulation (Atwal et al., 2000; Gupta et al., 2013) and that pro-BDNF further aggravate the issue by inducing neuronal cell death in complex with increased Sortilin levels (Teng et al., 2005; Yang et al., 2014; Zhou et al., 2018). Quantifications of p75NTR levels and measurements of Sortilin/p75NTR complex formation may shed light on this speculative model. In addition, miRNAs with more than 30% regulation, targeting *Trkb* (miR-103-1 and miR-212) both present significant upregulation in the brain of the WKY rats and could in part be responsible for the regulation of *Trkb* expression. Lastly, the mRNA stabilizing adenylate-uridylate (AU) rich element binding protein (AUBP) HuR was downregulated in the hippocampus at both the mRNA and protein levels indicating further mRNA decay in the WKY rats. In summary, compromised BDNF signaling, decreased mRNA stability (*TrkB*) and maturation of its encoded receptor could contribute to the depressive-like phenotype of WKY rats.

Lastly, as miRNAs are stably transported in blood and other fluids in exosomes or by lipoproteins, there has also been a considerable interest to identify peripheral miRNA changes associated with depression and antidepressant treatment in blood (Lopez et al., 2018). Here, the dysregulation of miRNAs in the brain does not translate into the blood as miR-122 and miR-206 exhibit robust upregulation in the blood but no regulation in the brain. This difference between brain and blood is a regular observation in the literature (Kaadt et al., 2021; Lopez et al., 2018). However, systemic alterations imposed by the depressive pathophysiology is likely resulting in local transcription in peripheral cells, which can lead to miRNA-alterations in blood, not found in the brain. It is evident that altered levels of miR-122 and miR-206 in serum has implications in renal carcinoma (Heinemann et al., 2018) and a large body of evidence identify miR-122 as a key regulator of liver development, homeostasis, and metabolic function, and elimination of miR-122 has been associated with liver disease (Bandiera et al., 2015). In accordance with our results, depression and liver disease has been linked in multiple studies, both clinically and mechanistically (Bianchi et al., 2005; Huang et al., 2017) and our observation of miR-122 regulation in the WKY rats may further add to this connection. Furthermore, we have recently identified miR-122-5p as a potential biomarker in plasma from depressed adolescents (Krivosova et al., 2023).

Not only in plasma, but also in the brain we identified WKY-regulated miRNAs in relation to studies including depressed patients. Five of the 13 specific miRNAs regulated across both brain regions in the WKY rats, have been associated with depression in humans, including miR-16 in serum (Gheysarzadeh et al., 2018), miR-20a (Smalheiser et al., 2012; Wang et al., 2018) and miR-195 in prefrontal cortex (Lopez et al., 2014), miR-92a in both plasma and prefrontal cortex (He et al., 2017; Wang et al., 2018), and miR-212 in serum (Lin et al., 2018). The overlap of the six miRNAs, identified in brain and blood, suggests that comparable regulatory pathways are at play across species. Moreover, regulation of miR-92a and miR-212 have been associated with antidepressant effects in rodent models of depression (Ji and Zhao, 2023). Ji et al. (2023) demonstrated that depressive-like behavior in rats induced by chronic unpredictable mild stress (CUMS) could be reversed by exposure to *enriched environment* (EE) and resulted in decreased levels of miR-92a in the hippocampus (Ji and Zhao, 2023). The effect of EE was blocked by Adeno-Associated Virus (AAV)-mediated overexpression of miR-92a, thus indicating that miR-92a acts in opposition to antidepressive treatment and might play a role in depression (Ji and Zhao, 2023). This agrees with the upregulation of miR-92a observed in the prefrontal cortex in the WKY rats. Furthermore, it has been demonstrated that ischemic stroke patients with high baseline levels of miR-92a-3p were more likely to develop early-onset post-stroke depression (He et al., 2017). However, miR-92a has also been reported to be downregulated in the prefrontal cortex of suicide completers (Wang et al., 2018) indicating there is not a clear consensus.

In parallel, Si et al. (2021) have demonstrated that depressive-like behavior exhibited by CUMS mice could be reversed by either Fluoxetine or over-expressing of miR-212 in the hippocampus (Si et al., 2021). Interestingly, it was reported that miR-212 already was upregulated in the hippocampus of the depressive-like CUMS mice, but that further overexpression of miR-212 resulted in the antidepressant and anti-inflammatory effect (Si et al., 2021). In accordance with the CUMS mice, we observed upregulation of miR-212 in the prefrontal cortex of the WKY rats. Cooperating these results, Ryan et al. (2023) demonstrated that rats subjected to electroconvulsive stimulation (ECS), the animal model equivalent to electroconvulsive therapy, resulted in upregulation of miR-212-3p (Ryan et al., 2023). In addition, Lin et al. showed that miR-212 was elevated in serum in from depressed patients after antidepressant treatment with selective serotonin re-uptake inhibitors (SSRIs) and serotonin and norepinephrine re-uptake inhibitors (SNRIs) (Lin et al., 2018). Nuclear factor I-A (NFIA) has been identified as the direct target for miR-212 which in turn regulates the level of the inflammatory factors TNF-α, IL-1β, and IL-6, which are generally associated with depression in human studies (Lee and Giuliani, 2019).

Finally, it is a limitation of the regulatory findings presented in Fig. 1, that the results have not undergone multiple test corrections and therefore are subject for further validation. However, as a whole, a considerable portion of the observed regulatory patterns maintain significance after correction for multiple testing (Tables S4–S8). Hence, in conclusion, this study identifies several molecular alterations in brain and blood from the WKY rats, which are linked to the depressive pathophysiology. We therefore further want to highlight the use of the WKY rats as a viable model of depression.

## CRediT author statement

Conceptualization: NKH, CKD, HKM, BE.

Methodology: NKH, CKD, HKM, BE.

Formal analysis: EK, NKH, BE.

Investigation: NKH, HKM, BE.

Writing – original draft: EK, NKH, BE.

Writing – review and editing: EK, CKD, HKM, BE.

Visualization: EK, BE.

Supervision: CKD, HKM, BE.

Funding acquisition: BE.

## Conflict of interest

The authors have no conflicts of interest to disclose. All authors have read the journal's authorship agreement and policy on disclosure of potential conflicts of interest. No editorial support was used in writing the manuscript. The manuscript has been reviewed and approved by all named authors.
