# Supplementary material for: From microRNA to protein, linking the neurotrophic hypothesis of depression to the Wistar Kyoto rat
Source: Neurosci Appl. 2023 Aug 19;2:101131. doi: 10.1016/j.nsa.2023.101131 (PMC12244101; doi:10.1016/j.nsa.2023.101131)
Supplement: Multimedia component 1 [file mmc1.docx]

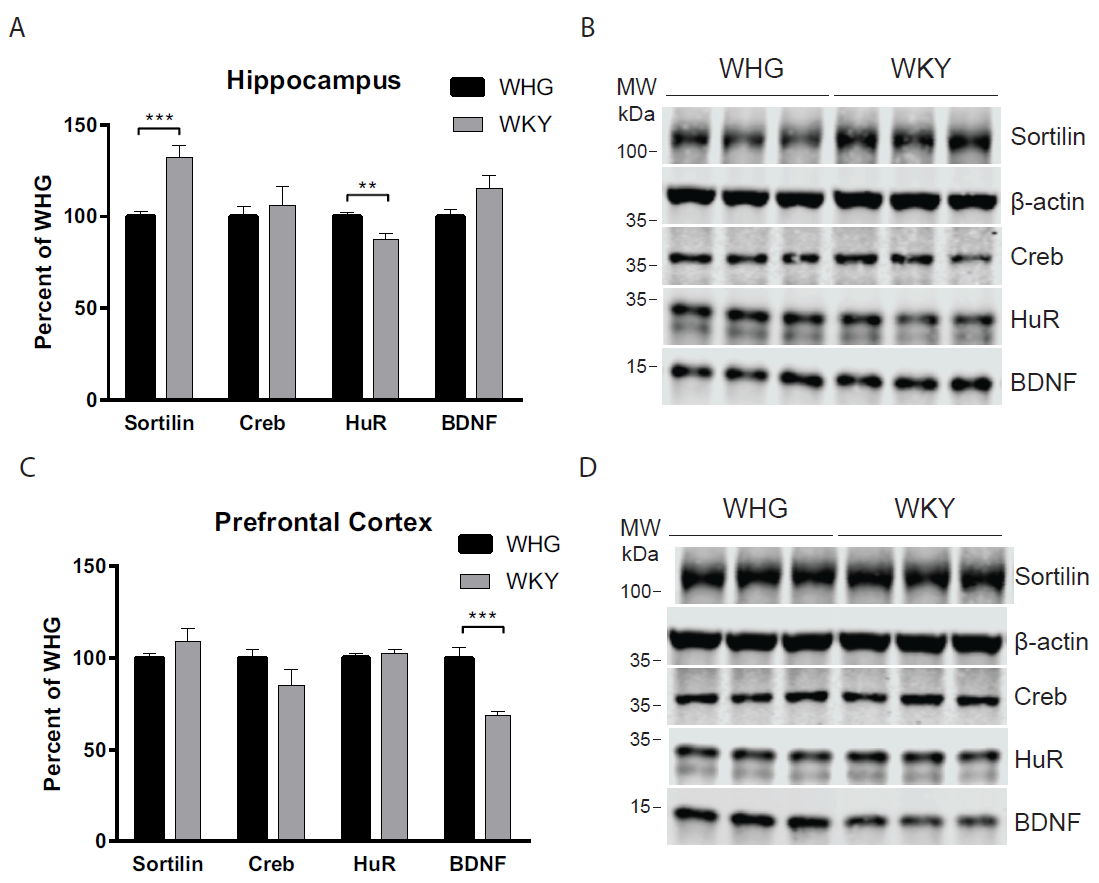


Figure S1. Immunoblotting results of Sortilin, HuR, Creb, and BDNF.

Bar graphs illustrating normalized protein levels for Sortilin, HuR, Creb, and BDNF in the WKY rats as percentage of the WHG rats in hippocampus (A, B) and prefrontal cortex (C, D). Representative blots are shown. Data are presented as mean + SEM. N=10 in each group, except WKY hippocampus n=9 (t-test, **p < 0.01, ***p < 0.001).


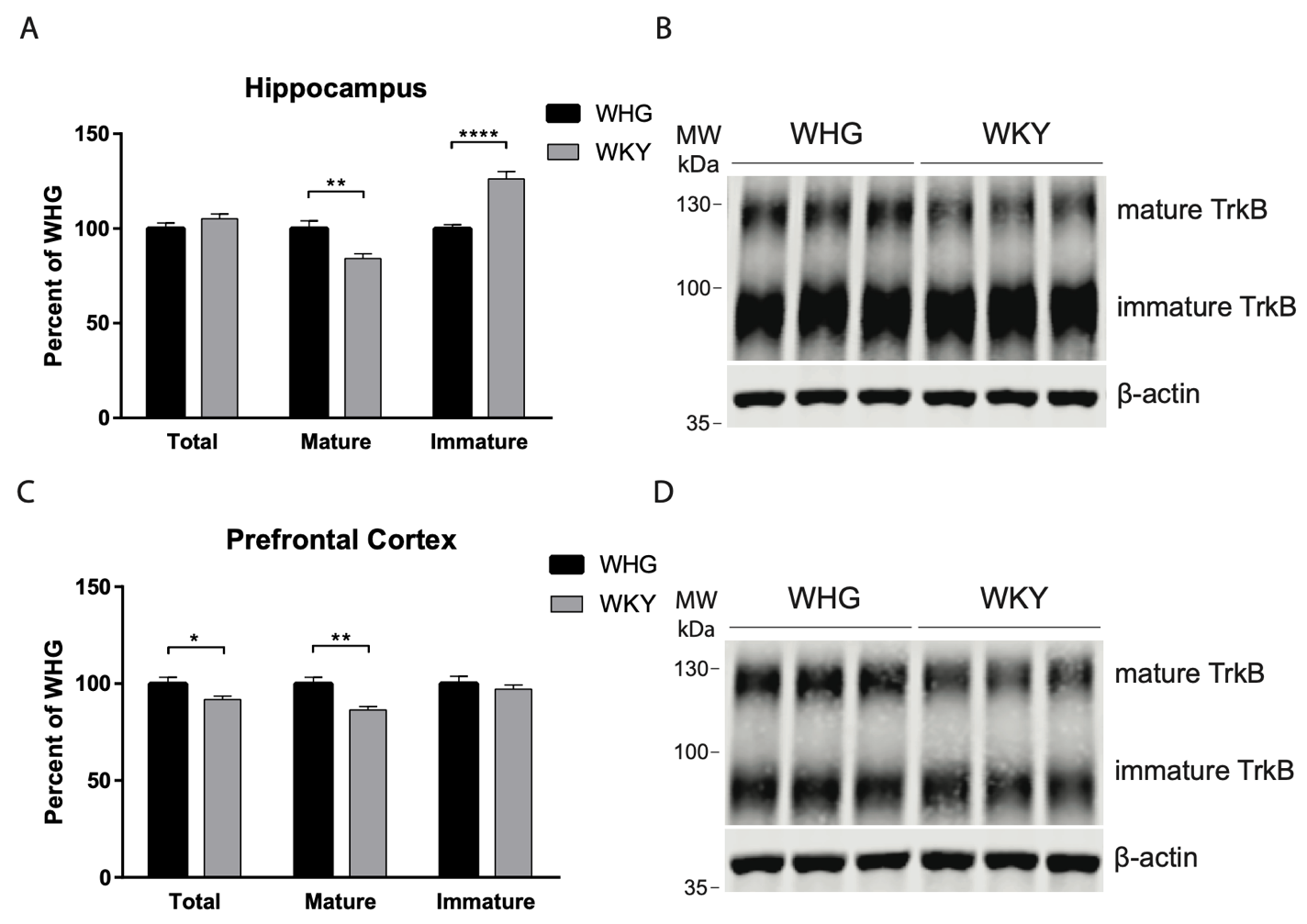


Figure S2. Immunoblotting results of Immature, Mature, and Total TrkB.

Bar graphs illustrating normalized protein levels for Immature, Mature, and Total TrkB in the WKY rats as percentage of the WHG rats in hippocampus (A, B) and prefrontal cortex (C, D). Representative blots are shown. Data are presented as mean + SEM. N=10 in each group, except WKY hippocampus n=9 (t-test, *p < 0.05, **p < 0.01, ****p < 0.0001).
