# Supplementary material for: From microRNA to protein, linking the neurotrophic hypothesis of depression to the Wistar Kyoto rat
Source: Neurosci Appl. 2023 Aug 19;2:101131. doi: 10.1016/j.nsa.2023.101131 (PMC12244101; doi:10.1016/j.nsa.2023.101131)
Supplement: Multimedia component 2 [file mmc2.docx]

| **Table S1. Relevant genes** | |
| --- | --- |
| **Gene** | **Relevance for depression or miRNA-function** |
| **Hur** | The efficacy of miRNAs is in part regulated by adenylate-uridylate (AU) rich element binding proteins (AUBPs). which bind to AU-rich elements. mostly located in the 3’ untranslated region of mRNAs^1-7^. Although AUBPs often regulate mRNA stability by enhancing or inhibiting the recruitment of the general mRNA decay machinery^8.9^. they can also function by recruiting or inhibiting miRNA function^1-7^. *Hur* is an mRNA stabilizing AUBP^3^. |
| cAMP response element-binding protein (**CREB**) | CREB promotes transcription of **Brain-derived Neurotrophic factor (BDNF)**. and phosphorylated CREB is regulated in parieto-occipital cortex and prefrontal cortex from depressed patients compared to controls^10^. |
| Tropomyosin-related kinase receptors (**TrkB + TrkC**) | **BDNF** exerts its function through binding to tropomyosin-related kinase B (TrkB) receptors^11^ and in depressed suicide victims a down-regulation of both mRNA and protein levels of BDNF and TrkB in prefrontal cortex have been identified^12^. Furthermore. reduced TrkB mRNA levels have been reported in subgenual anterior cingulate cortex from depressed patients compared to healthy controls^13^ demonstrating the dysfunction of this pathway in the brain of depressed patients.  TrkC is the receptor for the neurotrophic factor neurotrophin-3^14.15^. |
| matrix metalloproteinase 9 (**Mmp9**) | The precursor of **BDNF** (proBDNF) is enzymatically cleaved by either intracellular or extracellular proteases to mature BDNF (mBDNF). In particular. matrix metalloproteinase 2 and 9 (MMP2. MMP9) have been suggested as key players in depression^16.17^. Evidently. both MMP2 and MMP9 mRNA and protein levels are up-regulated in blood from depressed patients compared to healthy controls^16^ and in a previous profiling study. MMP9 has been suggested a candidate marker of depression^17^. |
| **Sortilin** | Sortilin plays a role in regulating **BDNF**^18-20^. and serum sortilin levels are increased in depressed individuals compared to controls. and correlate with the corresponding BDNF and Vascular Endothelial Growth Factor (VEGF) levels^21^. |

1 Kumar. A.. Varendi. K.. Peränen. J. & Andressoo. J. O. Tristetraprolin is a novel regulator of BDNF. *Springerplus* **3**. 502 (2014). <https://doi.org:10.1186/2193-1801-3-502>

2 Essafi-Benkhadir. K.. Onesto. C.. Stebe. E.. Moroni. C. & Pagès. G. Tristetraprolin inhibits Ras-dependent tumor vascularization by inducing vascular endothelial growth factor mRNA degradation. *Mol Biol Cell* **18**. 4648-4658 (2007). <https://doi.org:10.1091/mbc.e07-06-0570>

3 Kurosu. T. *et al.* HuR keeps an angiogenic switch on by stabilising mRNA of VEGF and COX-2 in tumour endothelium. *Br J Cancer* **104**. 819-829 (2011). <https://doi.org:10.1038/bjc.2011.20>

4 Lim. C. S. & Alkon. D. L. Protein kinase C stimulates HuD-mediated mRNA stability and protein expression of neurotrophic factors and enhances dendritic maturation of hippocampal neurons in culture. *Hippocampus* **22**. 2303-2319 (2012). <https://doi.org:10.1002/hipo.22048>

5 Kim. H. H. *et al.* HuR recruits let-7/RISC to repress c-Myc expression. *Genes Dev* **23**. 1743-1748 (2009). <https://doi.org:10.1101/gad.1812509>

6 Srikantan. S.. Tominaga. K. & Gorospe. M. Functional interplay between RNA-binding protein HuR and microRNAs. *Curr Protein Pept Sci* **13**. 372-379 (2012). <https://doi.org:10.2174/138920312801619394>

7 Kundu. P.. Fabian. M. R.. Sonenberg. N.. Bhattacharyya. S. N. & Filipowicz. W. HuR protein attenuates miRNA-mediated repression by promoting miRISC dissociation from the target RNA. *Nucleic Acids Res* **40**. 5088-5100 (2012). <https://doi.org:10.1093/nar/gks148>

8 Otsuka. H.. Fukao. A.. Funakami. Y.. Duncan. K. E. & Fujiwara. T. Emerging Evidence of Translational Control by AU-Rich Element-Binding Proteins. *Front Genet* **10**. 332 (2019). <https://doi.org:10.3389/fgene.2019.00332>

9 Damgaard. C. K. & Lykke-Andersen. J. Regulation of ARE-mRNA Stability by Cellular Signaling: Implications for Human Cancer. *Cancer Treat Res* **158**. 153-180 (2013). <https://doi.org:10.1007/978-3-642-31659-3_7>

10 Laifenfeld. D.. Karry. R.. Klein. E. & Ben-Shachar. D. Alterations in cell adhesion molecule L1 and functionally related genes in major depression: a postmortem study. *Biol Psychiatry* **57**. 716-725 (2005). <https://doi.org:10.1016/j.biopsych.2004.12.016>

11 Gupta. V. K.. You. Y.. Gupta. V. B.. Klistorner. A. & Graham. S. L. TrkB receptor signalling: implications in neurodegenerative. psychiatric and proliferative disorders. *Int J Mol Sci* **14**. 10122-10142 (2013). <https://doi.org:10.3390/ijms140510122>

12 Dwivedi. Y. *et al.* Altered gene expression of brain-derived neurotrophic factor and receptor tyrosine kinase B in postmortem brain of suicide subjects. *Arch Gen Psychiatry* **60**. 804-815 (2003). <https://doi.org:10.1001/archpsyc.60.8.804>

13 Tripp. A. *et al.* Brain-derived neurotrophic factor signaling and subgenual anterior cingulate cortex dysfunction in major depressive disorder. *Am J Psychiatry* **169**. 1194-1202 (2012). <https://doi.org:10.1176/appi.ajp.2012.12020248>

14 Lamballe. F.. Klein. R. & Barbacid. M. trkC. a new member of the trk family of tyrosine protein kinases. is a receptor for neurotrophin-3. *Cell* **66**. 967-979 (1991). <https://doi.org:10.1016/0092-8674(91)90442-2>

15 Tessarollo. L. *et al.* trkC. a receptor for neurotrophin-3. is widely expressed in the developing nervous system and in non-neuronal tissues. *Development* **118**. 463-475 (1993). <https://doi.org:10.1242/dev.118.2.463>

16 Bobińska. K.. Szemraj. J.. Czarny. P. & Gałecki. P. Expression and Activity of Metalloproteinases in Depression. *Med Sci Monit* **22**. 1334-1341 (2016). <https://doi.org:10.12659/msm.895978>

17 Domenici. E. *et al.* Plasma protein biomarkers for depression and schizophrenia by multi analyte profiling of case-control collections. *PLoS One* **5**. e9166 (2010). <https://doi.org:10.1371/journal.pone.0009166>

18 Teng. H. K. *et al.* ProBDNF induces neuronal apoptosis via activation of a receptor complex of p75NTR and sortilin. *J Neurosci* **25**. 5455-5463 (2005). <https://doi.org:10.1523/jneurosci.5123-04.2005>

19 Evans. S. F. *et al.* Neuronal brain-derived neurotrophic factor is synthesized in excess. with levels regulated by sortilin-mediated trafficking and lysosomal degradation. *J Biol Chem* **286**. 29556-29567 (2011). <https://doi.org:10.1074/jbc.M111.219675>

20 Vaegter. C. B. *et al.* Sortilin associates with Trk receptors to enhance anterograde transport and neurotrophin signaling. *Nat Neurosci* **14**. 54-61 (2011). <https://doi.org:10.1038/nn.2689>

21 Buttenschøn. H. N. *et al.* Increased serum levels of sortilin are associated with depression and correlated with BDNF and VEGF. *Transl Psychiatry* **5**. e677 (2015). <https://doi.org:10.1038/tp.2015.167>

| **Table S2. The 49 miRNAs included in the real-time qPCR experiments** | | | |
| --- | --- | --- | --- |
| **miRNA ID** | **Product number^a^** | **miRNA ID** | **Product number^a^** |
| Rno-miR-1-3p | 205104 | Rno-miR-146a-5p^b.c^ | 204688 |
| Hsa-miR-7-5p^b^ | 205877 | Rno-miR-181a-5p^b.c^ | 206081 |
| Rno-mir-7a-1-3p^b.c^ | 205573 | Rno-miR-185-5p^b.c^ | 206037 |
| Rno-miR-7a-2-3p^b^ | 2116436 | Rno-miR-190b-5p | 205110 |
| Rno-miR-10b-5p | 205499 | Rno-miR-191a-5p^b^ | 204306 |
| Rno-miR-16-5p^b.c^ | 205702 | Rno-miR-195-5p^b^ | 205869 |
| Rno-miR-18a-5p^c^ | 204207 | Rno-miR-200a-3p | 204707 |
| Rno-miR-20a-5p^b.c^ | 204292 | Rno-miR-200b-3p | 205111 |
| Rno-miR-20b-5p | 204755 | Rno-miR-200c-3p | 205505 |
| Rno-miR-29b-2-5p^b^ | 204208 | Rno-miR-203a | 205914 |
| Rno-miR-34a-3p | 2107928 | Rno-mir-203b-5p | 2108357 |
| Rno-miR-92a-3p^b.c^ | 205947 | Rno-miR-204-5p^b^ | 206072 |
| Rno-miR-93-5p^b.c^ | 204715 | Rno-miR-206^b.c^ | 206073 |
| Rno-miR-96-5p | 204417 | Rno-miR-210-3p^b.c^ | 204333 |
| Hsa-miR-103a-3p^b.c^ | 20406 | Rno-miR-211-5p^b^ | 205091 |
| Rno-miR-103-1-5p^b.c^ | 2114367 | Rno-miR-212-5p^b^ | 2100625 |
| Rno-miR-103-2-5p | 2100993 | Rno-miR-221-5p | 2116701 |
| Hsa-miR-122-5p^c^ | 205664 | Rno-miR-331-3p^b.c^ | 206046 |
| Hsa-miR-125a-5p^b.c^ | 204339 | Rno-miR-365a-3p^b.c^ | 204622 |
| Rno-miR-125b-5p^b.c^ | 205713 | Rno-miR-375^b.c^ | 204362 |
| Rno-miR-126a-3p^b.c^ | 204227 | Rno-miR-485-5p^b^ | 2112548 |
| Rno-miR-128-3p^b.c^ | 205995 | Rno-miR-497-5p^b^ | 205164 |
| Rno-miR-134-3p | 2114564 | Rno-miR-503-5p | 205094 |
| Rno-miR-135a-5p^b^ | 204762 | Rno-miR-628 | 205147 |
| Rno-miR-145-5p^b.c^ | 204483 |  |  |

^a^The product number from Exiqon (Denmark)

^b^The 31 miRNAs expressed in the brain

^c^The 22 miRNAs expressed in whole blood

**Table S3. Characteristics of gene-specific mRNA primers used for real-time qPCR**

| **Gene symbol** | **Gene name** | **Accession no.^a^** | **Primer sequence** | **Amplicon size^b^** |
| --- | --- | --- | --- | --- |
| **REFERENCE GENES** | |  |  |  |
| *18s rRNA* | 18s subunit ribosomal RNA | M11188 | (+) acggaccagagcgaaagcat  (-) tgtcaatcctgtccgtgtcc | 310 |
| *Actb* | Beta-actin | NM_031144 | (+) tgtcaccaactgggacgata  (-) ggggtgttgaaggtctcaaa | 165 |
| *CycA* | Cyclophilin A | XM_345810 | (+) agcactggggagaaaggatt  (-) agccactcagtcttggcagt | 248 |
| *Gapdh* | Glyceraldehyde-3-phosphate dehydrogenase | NM_017008 | (+) tcaccaccatggagaaggc  (-) gctaagcagttggtggtgca | 169 |
| *Hmbs* | Hydroxy-methylbilane synthase | NM_013168 | (+) tcctggctttaccattggag  (-) tgaattccaggtgagggaac | 176 |
| *Hprt1* | Hypoxanthine guanine phosphoribosyl transferase 1 | NM_012583 | (+) gcagactttgctttccttgg  (-) cgagaggtccttttcaccag | 81 |
| *Rpl13A* | Ribosomal protein L13A | NM_173340 | (+) acaagaaaaagcggatggtg  (-) ttccggtaatggatctttgc | 167 |
| *Ywhaz* | Tyrosine 3-monooxygenase/tryptophan 5-monooxygenase activation protein. zeta | BC094305 | (+) ttgagcagaagacggaaggt  (-) gaagcattggggatcaagaa | 136 |
| **TARGET GENES** | | | | |
| *Bdnf* | Brain-derived neurotrophic factor | NM_001270630 | (+) gaaagtcccggtatcaaaag  (-) cgccagccaattctctttttg | 187 |
| *Creb* | cAMP responsive element binding protein | NC_005108 | (+) cgtcatctgctcccactgta  (-) ccttcgtttttgggaatcag | 194 |
| *Hur* | ELAV like RNA binding protein 1 (Elavl1) | [NM_001108848.1](http://www.ncbi.nlm.nih.gov/entrez/viewer.fcgi?db=nucleotide&id=157818152) | (+) cagaccacaggtttgtccaga  (-) ctagcaggcgagtggtacag | 194 |
| *Mmp9* | matrix metalloproteinase 9 | [NM_031055.1](http://www.ncbi.nlm.nih.gov/entrez/viewer.fcgi?db=nucleotide&id=13591992) | (+) gcatctgtatggtcgtggct  (-) tgcagtgggacacatagtgg | 100 |
| *Sortilin* |  | NC_005101.4 | (+) ctgaccaacaatacgcacca  (-) agttctcgggaccaatagcc | 210 |
| *Trkb* | Neurotrophic receptor tyrosine kinase. type 2 | NM_012731.2 | (+) cctcgttggagaagatcaag  (-) cgtggtactccgtgtgattg | 221 |
| *Trkc* | Neurotrophic receptor tyrosine kinase. type 3 | [NM_001270656.1](http://www.ncbi.nlm.nih.gov/entrez/viewer.fcgi?db=nucleotide&id=397174821) | (+) agaggctagcagcatcagcag  (-) tgcattcaatgacctctgtgttaga | 243 |
| *Vegf* | Vascular endothelial growth factor A | NM_031836 | (+) aatgatgaagccctggagtg  (-) tttcttgcgctttcgttttt | 210 |

^a^ Genbank accession number of cDNA and corresponding gene available at <http://www.ncbi.nlm.nih.gov/>.

^b^ Amplicon length in base pairs.

| **Table S4. Investigated miRNAs in the hippocampus** | | | | | | | |
| --- | --- | --- | --- | --- | --- | --- | --- |
| **miRNA ID** | **WHG (%)**  **Mean (±SEM)** | **WKY (%)**  **Mean (±SEM)** | | ***t-test***  **P-value** | **Regulation** | **mRNA-targets** | **p-adjust**  **BH** |
|  | **Hippocampus** (normalized with miR-**125a-5p** and miR-**185-5p**) | | | | | | |
| miR-**7** | 100 (±5.7) | 126.2(±7.6) | 0.0126 | | ↑ (+26%) | *Creb, Vegf* | 0.0332 |
| miR-**7a-1** | 100 (±8.3) | 122.4 (±16.0) | 0.0108 | | ↑ (+22%) | *Bdnf* | 0.0348 |
| miR-**7a-2** | 100 (±7.6) | 101.5 (±12.1) | 0.9173 | |  | *Bdnf* | 0.9501 |
| miR-**16** | 100 (±6.3) | 129.6 (±8.3) | 0.0110 | | ↑ (+30%) | *Sert, Hur, Bdnf, Vegf* | 0.0319 |
| miR-**20a** | 100 (±6.0) | 155.6 (±8.7) | < 0.0001 | | ↑ (+56%) | *Vegf* | 7.00E-04 |
| miR-**29b-2** | 100 (±6.2) | 120.8 (±10.0) | 0.0524 | | ↑ (+21%) | *Mmp9* | 0.0844 |
| miR-**92a** | 100 (±4.3) | 93.6 (±7.3) | 0.3288 | |  | *Trkc* | 0.4146 |
| miR-**93** | 100 (±4.1) | 123.8 (±5.2) | 0.0011 | | ↑ (+24%) | *Vegf, Creb, Trkb* | 0.0080 |
| miR-**103a** | 100 (±5.2) | 92.7 (±5.7) | 0.3560 | |  | *Bdnf, Trkb, Trkc, Vegf, Creb* | 0.4130 |
| miR-**103-1** | 100 (±10.2) | 131.6 (±10.0) | 0.0401 | | ↑ (+32%) | *Trk, Trkc, Sortilin* | 0.0727 |
| miR-**125b** | 100 (±3.3) | 109.2 (±6.0) | 0.1987 | |  | *Sortilin, Trkc* | 0.2619 |
| miR-**126** | 100 (±5.5) | 134.8 (±10.7) | 0.0097 | | ↑ (+35%) | *Vegf* | 0.0402 |
| miR-**128** | 100 (±6.6) | 125.7 (±5.3) | 0.0072 | | ↑ (+26%) | *Trkc* | 0.0348 |
| miR-**135a** | 100 (±3.9) | 125.1 (±7.1) | 0.0062 | | ↑ (+25%) | *Sert* | 0.0360 |
| miR-**145** | 100 (±6.9) | 107 (±8.9) | 0.5397 | |  | *Bdnf* | 0.6020 |
| miR-**146a** | 100 (±5.0) | 86.5 (±4.8) | 0.0684 | |  | *Sortilin* | 0.0992 |
| miR-**181a** | 100 (±4.7) | 129.6 (±11.5) | 0.0385 | | ↑ (+30%) | *Creb* | 0.0744 |
| miR-**191a** | 100 (±8.5) | 122.4 (±6.3) | 0.0485 | | ↑ (+22%) | *Bdnf* | 0.0827 |
| miR-**195** | 100 (±6.7) | 172.8 (±6.1) | < 0.0001 | | ↑ (+73%) | *Bdnf, Vegf* | 6.50E-06 |
| miR-**204** | 100 (±4.6) | 124.4 (±7.1) | 0.0100 | | ↑ (+24%) | *Trkb, Bdnf* | 0.0363 |
| miR-**206** | 100 (±13.7) | 113.1 (±9.8) | 0.9495 | |  | *Bdnf* | 0.9495 |
| miR-**210** | 100 (±9.5) | 99.66 (±9.6) | 0.7394 | |  | *Bdnf* | 0.7942 |
| miR-**211** | 100 (±7.2) | 127.1 (±9.6) | 0.0360 | | ↑ (+27%) | *Creb* | 0.0746 |
| miR-**212** | 100 (±4.6) | 126.5 (±4.4) | 0.0006 | | ↑ (+27%) | *Creb, Trkb, Mmp9, Trkc* | 0.0058 |
| miR-**331** | 100 (±4.8) | 116.5 (±5.4) | 0.0357 | | ↑ (+17%) | *Trkc, Creb* | 0.0796 |
| miR-**365a** | 100 (±6.8) | 126.8 (±8.6) | 0.0248 | | ↑ (+27%) | *Creb* | 0.0599 |
| miR-**375** | 100 (±5.5) | 125.5 (±12.7) | 0.0657 | |  | *Hud, Creb, Bdnf, Vegf* | 0.1003 |
| miR-**485** | 100 (±4.3) | 110.1 (±9.7) | 0.3527 | |  | *Trkc* | 0.4262 |
| miR-**497** | 100 (±6.3) | 112.9 (±5.8) | 0.1481 | |  | *Vegf* | 0.205 |

All miRNAs investigated in the hippocampus from the WKY rats compared to the WHG rats (t-test. p<0.05). miRNAs regulated more than 30% are underlined. N=10 in each group. BH: Benjamini-Hochberg correction for multiple testing.

| **Table S5. Investigated miRNAs in the prefrontal cortex** | | | | | | |
| --- | --- | --- | --- | --- | --- | --- |
| **miRNA ID** | **WHG (%)**  **Mean (±SEM)** | **WKY (%)**  **Mean (±SEM)** | ***t-test***  **P-value** | **Regulation** | **mRNA-targets** | **p-adjust**  **BH** |
|  | **Prefrontal Cortex** (normalized with miR-**16-5p** and miR-**128-3p**) | | | | | |
| miR-**7** | 100 (±12.8) | 110.9 (±8.4 ) | 0.2475 |  | *Creb, Vegf* | 0.342 |
| miR-**7a-1** | 100 (±10.4) | 116 (±7.1) | 0.2340 |  | *Bdnf* | 0.339 |
| miR-**7a-2** | 100 (±7.0) | 139.3 (±6.8) | 0.0008 | ↑ (+39%) | *Bdnf* | 0.012 |
| miR-**20a** | 100 (±6.1) | 94.7 (±3.0) | 0.4511 |  | *Vegf* | 0.545 |
| miR-**29b-2** | 100 (±6.3) | 191.0 (±30.0) | 0.0082 | ↑ (+91%) | *Mmp9* | 0.026 |
| miR-**92a** | 100 (±9.7) | 154.2 (±13.2) | 0.0015 | ↑ (+54%) | *Trkc* | 0.009 |
| miR-**93** | 100 (±4.4) | 92.2 (±4.2) | 0.2188 |  | *Vegf, Creb, Trkb* | 0.334 |
| miR-**103a** | 100 (±6.2) | 72.0 (±5.1) | 0.0026 | ↓ (-28%) | *Bdnf, Trkb, Trkc, Vegf* | 0.009 |
| miR-**103-1** | 100 (±3.8) | 117 (±2.4) | 0.0009 | ↑ (+17%) | *Trkb, Trkc, Sortilin* | 0.013 |
| miR-**125a** | 100 (±7.2) | 94.4 (±11.7) | 0.5225 |  | *Hur, Vegf, Trkc* | 0.561 |
| miR-**125b** | 100 (±4.5) | 82.1 (±6.0) | 0.0334 | ↓ (-18%) | *Sortilin, Trkc* | 0.075 |
| miR-**126** | 100 (±8.0) | 69.9 (±7.3) | 0.0126 | ↓ (-30%) | *Vegf* | 0.033 |
| miR-**135a** | 100 (±7.8) | 71.5 (±6.4) | 0.0113 | ↓ (-29%) | *Sert* | 0.033 |
| miR-**145** | 100 (±7.5) | 148.3 (±12.8) | 0.0043 | ↑ (+48.3%) | *Bdnf* | 0.016 |
| miR-**146a** | 100 (±9.0) | 72.6 (±9.5) | 0.0498 | ↓ (-27%) | *Sortilin* | 0.096 |
| miR-**181a** | 100 (±7.6) | 61.3 (±4.0) | 0.0003 | ↓ (-39%) | *Creb* | 0.009 |
| miR-**185** | 100 (±8.3) | 82.5 (±4.6) | 0.0848 |  | *Trkb* | 0.145 |
| miR-**191a** | 100 (±13.3) | 102.6 (±8.6) | 0.6326 |  | *Bdnf* | 0.655 |
| miR-**195** | 100 (±12.3) | 86.3 (±7.7) | 0.3559 |  | *Bdnf, Vegf* | 0.449 |
| miR-**204** | 100 (±6.0) | 125.5.8 (±8.0) | 0.0169 | ↑ (+26%) | *Trkb, Bdnf* | 0.041 |
| miR-**206** | 100 (±21.1) | 100.2 (±7.3) | 0.5000 |  | *Bdnf* | 0.580 |
| miR-**210** | 100 (±12.2) | 99.2 (±8.1) | 0.8883 |  | *Bdnf* | 0.888 |
| miR-**211** | 100 (±9.2) | 166.8 (±17.6) | 0.0034 | ↑ (+67%) | *Creb* | 0.014 |
| miR-**212** | 100 (±7.2) | 149.7 (±8.2) | 0.0013 | ↑ (+50%) | *Creb, Trkb, Trkc* | 0.009 |
| miR-**331** | 100 (±7.0) | 123.1 (±11.5) | 0.1027 |  | *Trkc, Creb* | 0.165 |
| miR-**365a** | 100 (±9.1) | 76.4 (±6.8) | 0.0520 |  | *Creb* | 0.094 |
| miR-**375** | 100 (±8.2) | 213.1 (±48.9) | 0.0349 | ↑ (+113%) | *Hud, Creb, Bdnf, Vegf* | 0.072 |
| miR-**485** | 100 (±16.9) | 124.5 (±7.1) | 0.2488 |  | *Trkc* | 0.328 |
| miR-**497** | 100 (±3.90) | 96.6 (±3.4) | 0.5188 |  | *Vegf* | 0.579 |

All miRNAs investigated in the prefrontal cortex from the WKY rats compared to the WHG rats (t-test. p<0.05). miRNAs regulated more than 30% are underlined. N=10 in each group. BH: Benjamini-Hochberg correction for multiple testing.

| **Table S6. Investigated miRNAs in whole blood** | | | | | | |
| --- | --- | --- | --- | --- | --- | --- |
| **miRNA ID** | **WHG (%)**  **Mean (±SEM)** | **WKY (%)**  **Mean (±SEM)** | ***t-test***  **P-value** | **Regulation** | **mRNA-targets** | **p-adjust**  **BH** |
| **Whole blood** (normalized with miR-**20a-5p** and miR-**185-5p**) | | | | | | |
| miR-**7a-1** | 100 (±3.5) | 118.9 (±9.8) | 0.0751 |  | *Creb, Vegf* | 0.188 |
| miR-**16** | 100 (±9.7) | 93.2 (±7.8) | 0.5924 |  | *Sert, Hur, Bdnf, Vegf* | 0.658 |
| miR-**18a** | 100 (±4.9) | 104.4 (±9.1) | 0.6744 |  | *Trkb* | 0.710 |
| miR-**92a** | 100 (±9.5) | 109.5 (±7.9) | 0.4499 |  | *Trkc* | 0.643 |
| miR-**93** | 100 (±9.4) | 109.3 (±5.5) | 0.4069 |  | *Vegf, Creb, Trkb* | 0.626 |
| miR-**103a** | 100 (±6.8) | 78.7 (±3.5) | 0.0081 | ↓ (-21%) | *Trkb, Trkc, Sortilin* | 0.627 |
| miR-**103-1** | 100 (±14.7) | 112 (±7.0) | 0.4706 |  | *Trkb, Trkc, Sortilin* | 0.081 |
| miR-**122** | 100 (±22.5) | 244.2 (±36.1) | 0.0020 | ↑ (+144%) | *Creb, Bdnf* | 0.040 |
| miR-**125a** | 100 (±4.6) | 124.9 (±7.2) | 0.0094 | ↑ (25%) | *Hur, Vegf, Trkc* | 0.063 |
| miR-**125b** | 100 (±9.5) | 136 (±14.4) | 0.0518 |  | *Sortilin, Trkc* | 0.173 |
| miR-**126a** | 100 (±8.4) | 128.1 (±7.8) | 0.0152 | ↑ (28%) | *Vegf* | 0.076 |
| miR-**128** | 100 (±14.9) | 80.7 (±6.2) | 0.2460 |  | *Trkc* | 0.410 |
| miR-**145** | 100 (±5.6) | 116.8 (±7.5) | 0.0896 |  | *Bdnf* | 0.179 |
| miR-**146a** | 100 (±7.7) | 125.6 (±16.3) | 0.1728 |  | *Sortilin* | 0.314 |
| miR-**181a** | 100 (±8.1) | 92.7 (±7.0) | 0.4983 |  | *Creb* | 0.623 |
| miR-**206** | 100 (±26.1) | 234.2 (±47.5) | 0.0325 | ↑ (+134%) | *Bdnf* | 0.130 |
| miR-**210** | 100 (±9.8) | 135.8 (±16.9) | 0.0827 |  | *Bdnf* | 0.184 |
| miR-**331** | 100 (±6.5) | 94.5 (±5.5) | 0.5288 |  | *Trkc, Creb* | 0.622 |
| miR-**365a** | 100 (±11.9) | 98.5 (±6.0) | 0.9100 |  | *Creb* | 0.910 |
| miR-**375** | 100 (±12.4) | 192.7 (±48.1) | 0.0651 |  | *Hud, Creb, Bdnf, Vegf* | 0.186 |

All miRNAs measured in whole blood from the WKY rats compared to the WHG rats (t-test. p<0.05). miRNAs regulated more than 30% are underlined. N=10 in each group. BH: Benjamini-Hochberg correction for multiple testing.

| **Table S7. mRNA expression in the WKY compared to the WHG rats** | | | | | |
| --- | --- | --- | --- | --- | --- |
| **Gene** | **WHG (%)**  **Mean (± SEM)** | **WKY (%)**  **Mean (± SEM)** | ***t-test***  **P-value** | **Regulation** | **p-adjust**  **BH** |
|  | **Hippocampus** (normalized with *Hprt* and *CycA*) | | | | |
| Bdnf | 100 (±5.9) | 109.2 (±7.0) | 0.3237 | ↑ (+9.2%) | 0.370 |
| Creb | 100 (±4.3) | 91.5 (±4.5) | 0.1861 | ↓ (-8.5%) | 0.248 |
| Hur | 100 (±5.2) | 79.3 (±5.0) | **0.0101** | ↓ (-21%) | **0.027** |
| Mmp9 | 100 (±9.5) | 91.7 (±8.2) | 0.5179 | ↓ (-8.3%) | 0.5179 |
| Sortilin | 100 (±5.8) | 81.5 (±5.9) | **0.0377** | ↓ (-19%) | 0.0754 |
| Trkb | 100 (±4.5) | 79.1 (±3.4) | **0.0016** | ↓ (-21%) | **0.0128** |
| Trkc | 100 (±5.2) | 79.6 (±3.6) | **0.0045** | ↓ (-20%) | **0.018** |
| Vegf | 100 (±8.2) | 73.8 (±10.5) | 0.0644 | ↓ (-26%) | 0.103 |
|  | **Prefrontal Cortex** (normalized with *Hprt* and *Ywhaz*) | | | | |
| Bdnf | 100 (±12.6) | 90.5 (±5.4) | 0.4962 | ↓ (-9.5%) | 0.567 |
| Creb | 100 (±9.0) | 74.4 (±4.8) | **0.0212** | ↓ (-26%) | 0.057 |
| Hur | 100 (±13.4) | 76.6 (±3.2) | 0.1056 | ↓ (-23%) | 0.169 |
| Mmp9 | 100 (±9.4) | 98.1 (±8.7) | 0.8823 | ↓ (-1.9%) | 0.882 |
| Sortilin | 100 (±9.2) | 67.3 (±3.9) | **0.0060** | ↓ (-33%) | **0.048** |
| Trkb | 100 (±8.9) | 75.5 (±2.3) | **0.0160** | ↓ (-25%) | 0.064 |
| Trkc | 100 (±7.3) | 85.7 (±1.5) | 0.0717 | ↓ (-14%) | 0.143 |
| Vegf | 100 (±16.8) | 85.6 (±9.7) | 0.4673 | ↓ (-14%) | 0.623 |

All mRNAs measured in hippocampus and prefrontal cortex from the WKY rats compared to the WHG rats (t-test. p<0.05). N=10 in each group. BH: Benjamini-Hochberg correction for multiple testing.

| **Table S8. Protein expression in the WKY compared to the WHG rats** | | | | | |
| --- | --- | --- | --- | --- | --- |
| **Protein** | **WHG (%)**  **Mean (± SEM)** | **WKY (%)**  **Mean (± SEM)** | ***t-test***  **P-value** | **Regulation** | **p-adjust**  **BH** |
|  | **Hippocampus** | | | |  |
| Bdnf | 100 (±12.4) | 115.2 (±21.0) | 0.068 | ↑ (+15.2%) | 0.095 |
| Creb | 100 (±17.2) | 105.8 (±31.8) | 0.619 | ↑ (+5.8%) | 0.619 |
| Hur | 100 (±7.7) | 87.7 (±9.0) | **0.005** | ↓ (-12.3%) | **0.009** |
| Sortilin | 100 (±8.1) | 132.5 (±19.2) | **1.37E-04** | ↑ (32.5%) | **4.80E-04** |
| Trkb (total) | 100 (±9.1) | 105.2 (±7.7) | 0.201 | ↑ (5.2%) | 0.235 |
| Trkb (mat.) | 100 (±12.7) | 84.2 (±7.6) | **0.0048** | ↓ (-15.8%) | **0.011** |
| Trkb (imm.) | 100 (±6.7) | 126.2 (±11.8) | **1.29E-05** | ↑ (26.2 %) | **9.05E-05** |
|  | **Prefrontal Cortex** | | | |  |
| Bdnf | 100 (±19.2) | 68.7 (±6.8) | **3.19E-04** | ↓ (-31.3%) | **0.002** |
| Creb | 100 (±15.6) | 85.0 (±28.0) | 0.289 | ↓ (-15%) | 0.506 |
| Hur | 100 (±7.8) | 102.7 (±6.9) | 0.566 | ↑ (2.7%) | 0.566 |
| Sortilin | 100 (±8.2) | 109.2 (±22.3) | 0.394 | ↑ (9.2%) | 0.552 |
| Trkb (total) | 100 (±10.6) | 91.8 (±5.4) | **0.0497** | ↓ (-8.2%) | 0.116 |
| Trkb (mat.) | 100 (±10.4) | 86.5 (±5.3) | **0.0022** | ↓ (-13.5%) | **0.008** |
| Trkb (imm.) | 100 (±11.9) | 97.2 (±6.6) | 0.550 | ↓ (-2.8%) | 0.642 |

An overview of the proteins investigated in hippocampus and prefrontal cortex from the WKY rats compared to the WHG rats (t-test. p<0.05). N=10 in each group, except WKY hippocampus n=9. BH: Benjamini-Hochberg correction for multiple testing.
